# Genotyping of Fresh and Parafinized Human Hydatid Cysts Using *nad1* and *cox1* Genes in Hamadan Province, West of Iran

**Published:** 2020

**Authors:** Ali Ehsan SHAHBAZI, Massoud SAIDIJAM, Amir Hossein MAGHSOOD, Mohammad MATINI, Moosa MOTAVALI HAGHI, Mohammad FALLAH

**Affiliations:** 1. Department of Medical Parasitology and Mycology, School of Medicine, Hamadan University of Medical Sciences, Hamadan, Iran; 2. Department of Molecular Medicine and Genetics, School of Medicine, Hamadan University of Medical Sciences, Hamadan, Iran

**Keywords:** *Echinococcus granulosus*, genotypes, *nad1* protein, *cox1* protein, Iran

## Abstract

**Background:**

Hydatidosis is a cosmopolitan zoonotic infection and Hamadan Province in the west of Iran is one of the most important foci of human hydatidosis in Iran. The aim of the current study was the genetic characterization of hydatid cysts operated from humans in Hamadan Province.

**Methods:**

Seventy-two hydatid cysts samples including 50 paraffinized and 22 fresh human hydatid cysts collected from different hospitals in Hamadan Province, western Iran. The cysts' DNA genome was extracted by kit and PCR was performed for amplifying the fragments of 400 and 450bp for *nad1* and *cox1* mitochondrial genes, respectively. Genotype diversity and sequence variations of the cysts' isolates were studied by related software.

**Results:**

DNA from all (100%) paraffinized and fresh hydatid cysts samples extracted successfully. All paraffinized and fresh hydatid cysts samples were amplified by PCR assay using *nad1*gene, however, only 18 and 8 samples from paraffinized and fresh hydatid cyst samples was amplified using *cox1* gene, respectively. The sequences analysis indicated that, 98.61% the *Echinococcus granulosus* samples were belong to the genotype G1 and 1.39% were G3 genotype.

**Conclusion:**

Genotypes of *E. granulosus* in human samples in Hamadan Province are G1 and G3 and these findings are proved by phylogenic analysis.

## Introduction

Hydatidosis is a parasitic disease that may involve various organs and is still a common parasitic zoonosis which causes remarkable mortality (1,2). The higher prevalence of hydatidosis in human and animal hosts is reported in sheep-raising countries. This disease is one of the most frequently zoonotic infections in different regions of Iran with the incidence rate of 0.1 to 4.5/100,000 cases (3,4).

Different genotypes (G1 to G10) of this parasite have been reported from different origins and survey on the dominant genotypes in each region can play an essential role in determining of its control strategies (5). In spite of the Hamadan Province is one the hyper-endemic areas for hydatid disease but, there is little information about the genotypes of the human hydatid cysts in this region (6, 7). The prevalence of human hydatidosis was evaluated based on hospital records in Hamadan and reported that hydatidosis is a major health problem in this region (8), but there has not been a comprehensive molecular study on determining the dominant genotypes of the parasite in humans. Because the main occupation of this province inhabitants' are livestock farming and close relationship between people and definitive host of parasite and the risk of transmission of the disease, the predominant genotype of the parasite in this region and the source of the disease should be determined.

In this study, cytochrome c oxidase I (*cox1*) and NADH dehydrogenase subunit I (*nad1*) as target genes were used to identification of genotypes of larval stage *E. granulosus* from humans in Hamadan Province.

## Materials and Methods

### Climatic situations of the sampling area

Hamadan Province is in the west of Iran located in the northern slope of Zagrus Mountains, a major sheep rising area with Mediterranean climate the average altitude of these areas is about 1330 m. The province lies between 59° and 33′ to 49° and 35′ north latitude and 34° and 47′ to 34° and 49′ east of the Greenwich meridian.

The maximum absolute temperature in this Province is 36.8 °C and the minimum absolute is −29.6 °C and the average temperature is 9.6 °C. The hottest months of the year, with a maximum temperature of 35 °C, are Jul and Aug, and the coldest months of the year, with an average of −25.5 °C at Feb (9).

### Sampling

Overall, 72 hydatid cysts (50 paraffinized and 22 fresh cysts) were collected from patients who underwent surgery for hydatidosis from Besat and Fatemieh University Hospitals. In the fresh operated cysts, the fertility of cysts was determined using microscopic examination of the hydatid content. The cyst’s fluid was transferred into the test tube and left to set for 30 min. The supernatant was removed and the remained protoscoleces were washed 3 times with normal saline and stored at 4 °C until use. Germinal layer from operated cysts also used for DNA extraction.

**Fig. 1:**
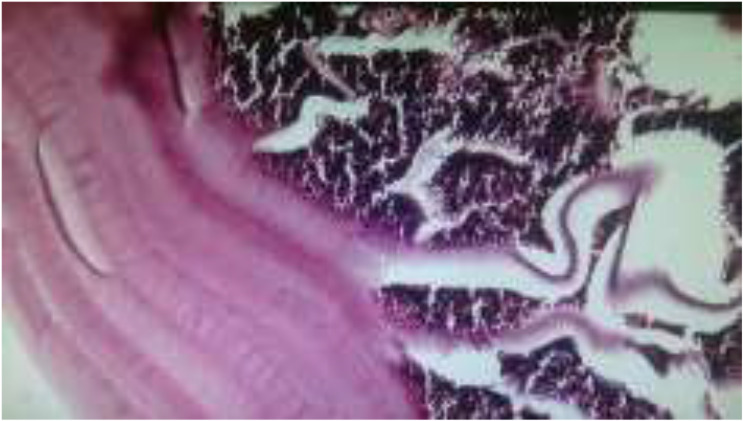
Laminated collagenous layer, hydatid cyst (X1000)

Moreover, paraffinized confirmed hydatid cysts were collected from different hospitals in Hamadan Province. Serial sections from all tissue samples were prepared and hydatid cysts re-confirmed by microscopic study by an expert pathologist (Fig. 1), then samples were used for DNA extraction.

### DNA extraction

DNA extraction from paraffinized tissue and fresh hydatid cyst samples was performed by a commercial kit (Qiagen DNA FFPE tissue kit for the paraffinized sample and QIAamp DNA Mini Kit for fresh hydatid cyst fluids, Germany) according to the manufacturer’s instructions. All extracted DNA stored at −20 °C until use (10).

### PCR assay

The targeted DNA regions were amplified for the *cox1* gene by conventional PCR method. The conventional PCR was performed in total volume 50 μl including: Tris-HCL (pH 9.0) (10 mM), KCl (500 mM), MgCl2 (2.5 mM), DNA template (200 ng), each dNTP (dATP, dCTP, dGTP, dTTP) (250 μM), Taq DNA polymerase (1U) and 20 pmol of, JB3 forward: 5'-TTTTTTGGGCATCCTGAGGTTTAT -3' and JB4.5 reverse: 5'-TAAAGA AAG AAC ATA ATG AAA ATG -3' to amplify a 450 bp fragment of *cox1* gene under the following conditions: 5 min at 95 °C, followed by 30 cycles of 95 °C for 60 sec, 55 °C for 60 sec and 72 °C for 1 min, with a final extension step at 72 °C for 5 min. And for *nad1* gene, MS 1, forward: (5'-CGTAGGTATGTTGGTTTGTTTGGT-3') and MS2, reverse: (5'-CCATAATCAAATGGCGTACGAT -3') primers were used to amplify a 400 bp fragment of *nad1* gene (11). PCR cycles were set up for 5 min at 95 °C, followed by 30 cycles of 95 °C for 60 sec, 55 °C for 60 sec and 72 °C for 1 min, with a final extension step at 72 °C for 5 min. The PCR products were electrophoresed in 1.5% agarose gel and stained by ethidium bromide. The PCR products for each separate isolate were sequenced by Bioneer Sequencing Service (South Korea) and the results of sequencing were Blast (http://blast.ncbi.nlm.nih.gov/Blast.cgi ) and the sequences were compared with those mitochondrial DNA sequences that already deposited in Genbank (12). Phylogenic analysis was done using the Maximum Likelihood method based on the Tamura-Nei model in MEGA 5.0 software and the nucleotide sequence of the cattle strain of *E. granulosus* (GenBank accession number KT988113) was used as outgroup.

This project approved with grant number 9604132296 and research ethics code: IR.UMSHA.REC.1396.27.

### Statistical analysis

Data was analyzed by SPSS software (ver. 22, Chicago, IL, USA). The descriptive statistics methods determine the frequency of the variables considered for main objectives of this research.

## Results

Seventy-two hydatid cysts, including 50 paraffinized and 22 fresh and newly operated human cyst samples, were subjected to genotyping of *E. granulosus* using the *nad1* and *cox1* genes. From all samples, 49 (68.06%), 19 (26.38%) and 4 (5.56%) were from liver, lung and spleen, respectively. Overall, 43 (59.73%) of cysts were from male and 29 (40.27%) were from female patients.

### PCR amplification

All paraffinized and fresh hydatid cysts samples were amplified by PCR assay using *nad1*gene, however, only 18 and 8samples from paraffinized and fresh hydatid cysts was amplified using *cox1* gene, respectively.

**Fig. 2:**
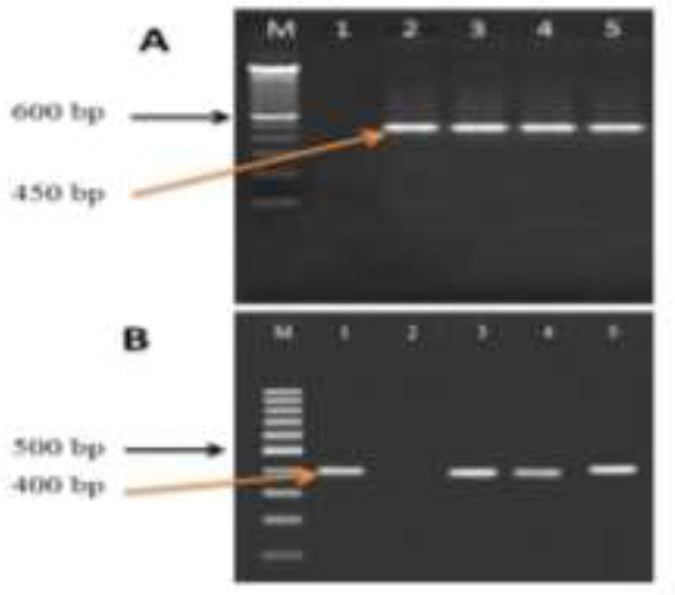
Agarose gel (1.2%), electrophoresis, *nad1* and*cox1* PCR products of *E. granulosus* derived from human paraffinized and fresh samples. A; Lane M: 100bp marker, line 1 negative control, line 2 positive control for cox1 gene, lines 3–5 positive sample for E*. granulosus.* B; Lane M: 100bp marker, line 1 positive control for *nad1* gene, line 2 negative control, lines 3–5 positive samples for E*. granulosus*

PCR amplification of the *nad1*and *cox1* genes yielded an expected 400 and 450bp fragment for DNA samples of hydatid cysts (Fig. 2).

Sequencing the PCR products confirmed that majority of the samples are same genotype and has 100% similarity with genotype G1 and only one human sample was genotype G3.

### Sequencing analysis

Sequence analysis was used for genotyping on the PCR products of *E. granulosus* sequenced using forward primer. Sequence legislatures for each of the recognized genotypes were submitted to the Gene Bank/EMBL/DDBJ database with accession number MK530690 to MK530693.

Sequence analysis of the *cox1* and *nad1* sequences determined that only one isolate belong to G3 genotype and the others belonged to *E. granulosus* G1. The G1 genotype isolates had one sequence type for *cox1* and *nad1* sequences.

### Phylogenic analysis

**Fig. 3:**
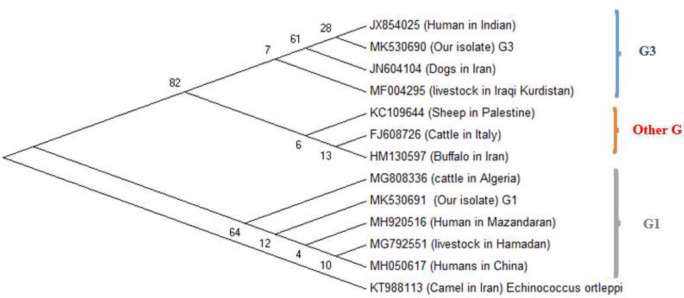
Phylogenic tree of haplotypes of *E. granulosus* samples from west of Iran. The phylogenic tree was constructed using the Maximum Likelihood method based on the Tamura-Nei model in MEGA5 software and bootstrap analysis with 1,000 re-samplings. Partial DNA sequences of concatenated mitochondrial *cox1* gene were used as input data. The -cattle strain of *E. granulosus* is KT988113 considered as outgroup strain

Sequence information was obtained from the NCBI database for characterization of isolates of *E. granulosus* isolates. The ClustalW2 software was applied to align the sequences and results were analyzed and compared with similar *E. granulosus* sequences deposited in GenBank using the Basic Local Alignment Search Tool (BLAST) program. Based on *cox1* gene sequences, a phylogenic tree was constructed using the Maximum Likelihood method based on the Tamura-Nei model in MEGA5 software and bootstrap analysis with 1,000 resampling (Fig. 3).

## Discussion

This study confirmed almost all samples belonged to same genotype, G1, and other genotypes reported in Iran have few importance in infecting humans. Consequently, the basic measures on the disrupting parasite life cycle have to concentrate on the dog-sheep cycle. Different strains of *E. granulosus* have significant differences in terms of morphological, biochemical, physiological and pathogenic aspects in different hosts (13). These differences can play an essential role in disease control planning and treatment. Several criteria have been used to identify the strains of the *E. granulosus* species. In Iran, *E. granulosus* strains were studied with morphological and biological methods on many parasite isolates. Subsequently, a total of 16 isolates of sheep, goat, camel, and human origin were studied by molecular methods (14). Hamadan Province is one of the endemic areas for echinoccocosis/hydatidosis in Iran, not yet studied the genotypes of human origin of *E. granulosus* comprehensively*.*

In an investigation, 74 animal isolates including 69 sheep, 3 cattle and 2 goats and 9 human hydatid cysts were genotyped by PCR amplification of the rDNA ITS1 region and followed by RFLP analysis with four restriction endonuclease enzymes. According to the RFLP patterns, the isolates belonged to a single species, *E. granulosus* (G1–G3 complex). Furthermore, sequencing of representative amplicons confirmed that the RFLP-genotyped isolates correspond to *E. granulosus* sensu stricto (15).

Because of the livestock farming in Hamadan Province, the rate of infection of animal hosts is relatively high, but there is no complete information about the genetic diversity of isolates from human (8,9). However, alignment of the obtained sequences with the reference sequence revealed 99% homology and the isolates corresponded to *E. granulosus* genotype G1 (15). Another study from neighbor province of Hamadan, Lorestan Province, determined all of sheep isolates (100%) belong to G1 and 88% of isolates from cattle also was G1, however only 11% of cattle isolates belong to G3 genotype (16). In another study in this province, 26 isolates from human patients were analyzed using mitochondrial *cox1* primers and sequencing demonstrated the isolates belonged to genotype G1(17).

Based on our knowledge, G1, G3 and G6 more often are reported from different parts of Iran. Hydatid cyst isolates were collected from human in Isfahan, Iran; amplification of ITS-1 region of rDNA and RFLP using AluI and MspI enzymes, the genotypes of 30 samples were determined. G1 was the dominant genotype of hydatid cyst extracted from different organs including liver, lung, and brain in this region (18).

In the another study on the 4 human and 30 animal hydatid cysts from the Ilam Province, west of Iran, by DNA genome of protoscoleces and RLFP-PCR using TaqI, HpaII, RsaI and AluI restriction enzymes; the results confirmed genotypes G1 and G3 in Ilam Province (19).

The results of current study indicated that most of isolates from human echinococcosis were G1 strain and only one G3 strain was found. A meta-analysis study reported that G1 is the most dominant genotype of *E. granulosus* in animals in Iran (20). Moreover, in some similar studies, in different provinces of Iran, the main genotype in all human isolates examined were G1 (21). Alternatively, there are few reports for G3 genotype in human infection.

Several nuclear and mitochondrial gene targets have formerly been practical effectively in distinction of these genotypes (11). Using the both genes; most genotypes in current work are allocated to the G1 genotype. In a similar work, Rostami Nejad et al. examined diversity of genotype using three mitochondrial genes *cox1*, *nad1* and *atp6* and also partial sequences of the 12S rRNA gene and reported that G1 and G6 genotypes are the most prevalent genotypes in different districts of Iran (22). Due to the weakness of the obtained band of the PCR product and their poor quality for the *cox1* gene sequencing, only 26 samples yielded desirable amplified cox1 gene.

Phylogenic analysis based on *cox1* indicated that humans G1 genotype from Hamadan Province have 95% to 100% homology with G1 genotype of Mazandaran, Hamadan livestock, human isolates from China and Algiers (15, 23). G3 genotype is also found in current work that had 100% homology with G3 genotype from dogs isolates in Iran, cows isolates from Kurdistan territory, Iraq, and human isolates from India. Based on the phylogenic study the G1 and G3 genotypes from current study are allocated in two different casts that a cluster includes sheep genotype of Palestine, the cattle from Italy and Buffalo from Iran is allocated between them.

The primary goal of this study was the use of the sequencing of both the *nad1* and *cox1* genes, but the PCR product of 48 samples did not have a good quality for amplifying the *cox1* gene, and the electropherograms’ resulting from their sequencing were not suitable, therefore, only 26 samples from the cox1 gene were used to the sequencing and confirmation of the results of the *nad1* gene as well as the differentiation of a sample detected with the *nad1* marker, genotype G2 / G3. The specimen was detected in the sequencing of the *cox1* gene, confirmed as the genotype G3.

The most of the researchers use one or more genetic markers for the genotyping of *E. granulosus* simultaneously. Most commonly used markers include ITS1, cox1, nad1 or 12S rRNA. Genetic markers are useful in genotyping of *E. granulosus* and confirm the results of each other, although some markers are more distinct than others to genotyping for this parasite. These researchers use optionally more than one marker to increase the accuracy of the results and better differentiation of identified genotypes. In the present study, the genetic markers of *nad1* and *cox1* were simultaneously used to distinguish the G2/G3. The results of this study, like other studies, showed that these two genetic markers confirm the results of each other, although the power of differentiation of the marker *cox1* is greater than the *nad1* marker.

## Conclusion

G1 was the most common genotype among our isolates. Finally, due to the high genetic similarities that exist between human genotypes in Hamadan and those from sheep and dogs from other parts of Iran, it can be imagined for the transmit of *E. granulosus* in Hamadan of this cycle.
